# Sustained acoustic medicine for the treatment of musculoskeletal injuries: a systematic review and meta-analysis

**DOI:** 10.1186/s13102-021-00383-0

**Published:** 2021-12-18

**Authors:** Sandra L. Winkler, Anthony E. Urbisci, Thomas M. Best

**Affiliations:** 1 James A. Haley Veteran Administration Hospital, Tampa, FL USA; 2 UHealth Sports Medicine Institute, University of Miami, Coral Gables, FL USA

## Abstract

**Background:**

Musculoskeletal injuries account for 10 million work-limited days per year and often lead to both acute and/or chronic pain, and increased chances of re-injury or permanent disability. Conservative treatment options include various modalities, nonsteroidal anti-inflammatory drugs, and physical rehabilitation programs. Sustained Acoustic Medicine is an emerging prescription home-use mechanotransductive device to stimulate cellular proliferation, increase microstreaming and cavitation in situ, and to increase tissue profusion and permeability. This research aims to summarize the clinical evidence on Sustained Acoustic Medicine and measurable outcomes in the literature.

**Methods:**

A systematic literature review was conducted using PubMed, EBSCOhost, Academic Search Complete, Google Scholar and ClinicalTrials.gov to identify studies evaluating the effects of Sustained Acoustic Medicine on the musculoskeletal system of humans. Articles identified were selected based on inclusion criteria and scored on the Downs and Black checklist. Study design, clinical outcomes and primary findings were extracted from included studies for synthesis and meta-analysis statistics.

**Results:**

A total of three hundred and seventy-two participants (372) were included in the thirteen clinical research studies reviewed including five (5) level I, four (4) level II and four (4) level IV studies. Sixty-seven (67) participants with neck and back myofascial pain and injury, one hundred and fifty-six (156) participants with moderate to severe knee pain and radiographically confirmed knee osteoarthritis (Kellgren–Lawrence grade II/III), and one hundred forty-nine (149) participants with generalized soft-tissue injury of the elbow, shoulder, back and ankle with limited function. Primary outcomes included daily change in pain intensity, change in Western Ontario McMaster Osteoarthritis Questionnaire, change in Global Rate of Change, and functional outcome measures including dynamometry, grip strength, range-of-motion, and diathermic heating (temperature measurement).

**Conclusion:**

Sustained Acoustic Medicine treatment provides tissue heating and tissue recovery, improved patient function and reduction of pain. When patients failed to respond to physical therapy, Sustained Acoustic Medicine proved to be a useful adjunct to facilitate healing and return to work. As a non-invasive and non-narcotic treatment option with an excellent safety profile, Sustained Acoustic Medicine may be considered a good therapeutic option for practitioners.

## Key points

- The significant and clinically meaningful reduction in musculoskeletal pain (1.96–3.94 points, 0–10 point pain scales) and improvement in joint function (20–87%) were reported in n = 9 and n = 6 studies, respectively on Sustained Acoustic Medicine.
- In addition to measurable clinical outcomes reported in the literature, n = 2 studies reported on vigorous therapeutic heat (Δ4 °C to Δ12 °C) and n = 1 study on biological clearance of lactic acid from Sustained Acoustic Medicine treatment in human participants.
- Clinical evidence, health economic cost effectiveness and health provider positive opinions on Sustained Acoustic Medicine support treatment utilization in musculoskeletal conditions such as Osteoarthritis, tendinopathy, and myofascial pain.

## Introduction

Musculoskeletal pain is a common issue experienced by most of the general population at some point over the lifetime [1]. Chronic musculoskeletal pain affects 20–33% of the world population, approximately 1.71 billion people [2]. Musculoskeletal pain is defined as acute or chronic pain affecting bones, muscles, tendons, ligaments, and nerves. Chronic pain can significantly affect daily activities, quality of life while promoting disability resulting in staggering health costs. It is estimated that the US spends $240 billion annually on musculoskeletal pain-related medical care. Back pain is the most common musculoskeletal pain [3–5]. Approximately 70–80% of Americans will experience back pain in their lifetime. Back pain is the fifth leading cause of hospitalization [4, 5]. Chronic musculoskeletal pain is most common in the older population. Osteoarthritis, a significant cause of joint pain, affects more than one-third of people above age 60 [6]. Musculoskeletal pain is also highly prevalent in athletes and military personnel dealing with strains, sprains, and fractures [7].

Musculoskeletal pain can be caused by a variety of conditions including maximal or submaximal concentric contractions, joint contractures, and direct trauma, leading to the abnormal release of acetylcholine resulting in increased tension, blood flow restriction, inflammation, and tissue damage [8–13]. A combination of pharmacological and nonpharmacological intervention is used to treat musculoskeletal pain [14, 15]. Typical pharmacological regimens include nonsteroidal anti-inflammatory drugs (NSAIDs), opioids, and adjunctive analgesics. The long-term use of NSAIDs has adverse systemic effects [16–19]. The use of opioids is short to medium-term in pain treatment with the significant danger of addiction and potential overuse leading to death [20, 21]. Further, adjuvant analgesics including anticonvulsants, anti-depressants, and anxiolytics are increasingly used for chronic musculoskeletal pain [22, 23]. Nonpharmacological approaches include physical modalities, cryotherapy, heat therapy, therapeutic exercises, and acupuncture frequently coupled to medication usage [24–36].

Recently, noninvasive nonpharmacological treatments such as transcutaneous nerve stimulation therapy (TENS), laser, and ultrasound therapy have been added to treatment regimens as standalone or adjunctive therapies [37–47]. TENS acts through inhibition of Aβ-fibers activated pain [42, 43], laser therapy actives cellular metabolism, increasing growth factor production and matrix production. Ultrasound therapy mechanically and thermally actives the targeted tissue to modulate pain [37, 41, 46–49].

**Fig. 1:**
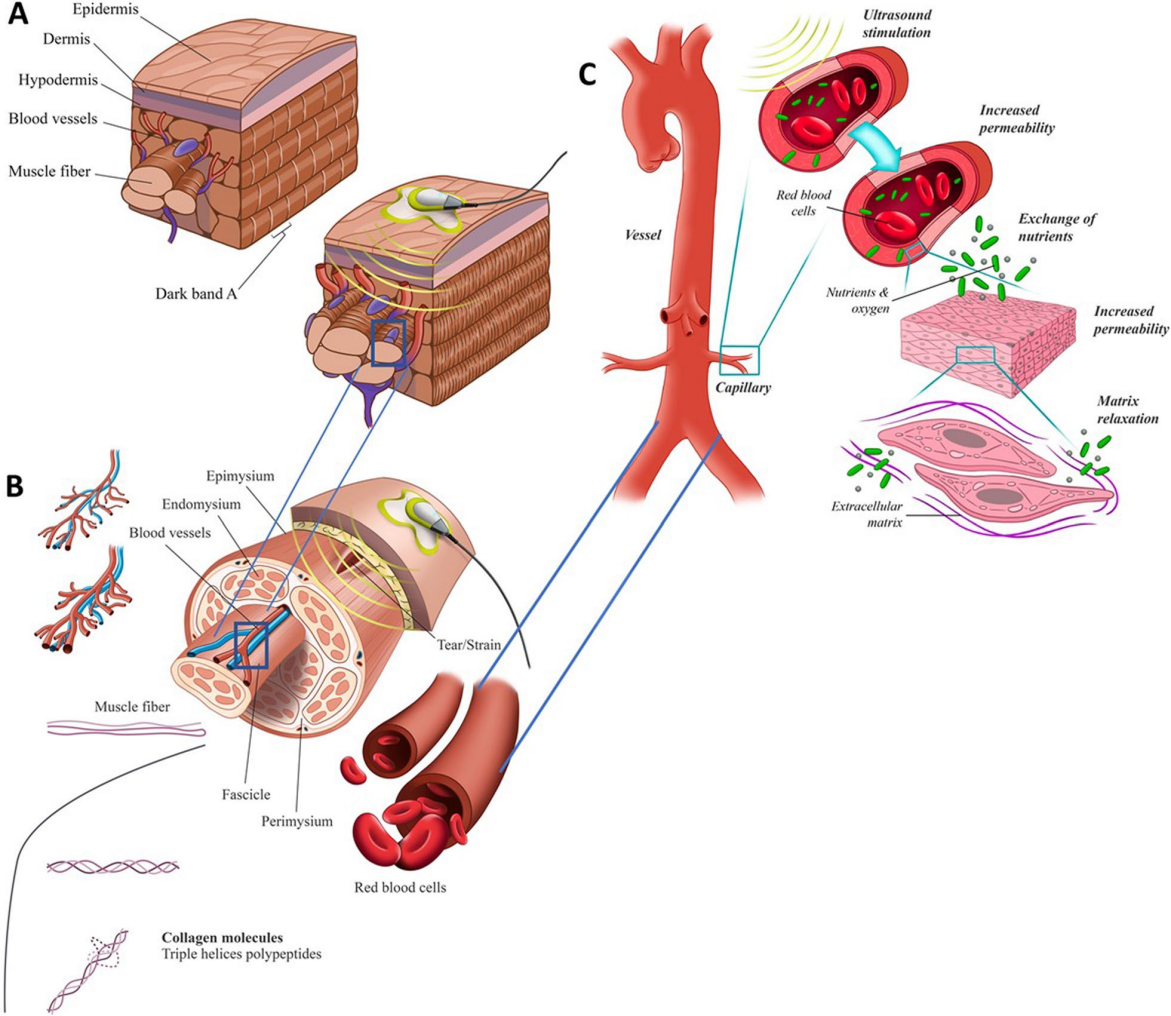
**A** Ultrasound increases cellular proliferation, tissue regeneration, and vascularity. These mechanisms are active daily over 4 h to upregulate healing, reduce inflammation and pain. **B** Ultrasound increases capillary permeability, increase nutrient exchange, oxygenation, and matrix relaxation at the site of injury, **C** ultrasound increases the vasodilation (vessels diameter), oxygenation, blood flow, and extends collagen fibers matrix

The Food and Drug Administration (FDA) in March 2020 approved Sustained Acoustic Medicine (SAM, ZetrOZ System LLC, FDA 510(k) #K191568, Class II, Medical Device) for prescription home use to treat pain, increase local circulation and improve joint function [49]. SAM utilizes high-frequency, low-intensity continuous ultrasound at 3 MHz with 0.132 mW/cm^2^ intensity delivering 18,720 J over 4 h of the treatment [50–52]. The SAM device allows for the long duration delivery of ultrasound stimulation to facilitate the healing of injured musculoskeletal tissue in the home of the patient [50, 53, 54]. SAM has mechanotransductive and diametric effects at the tissue and molecular level utilizing acoustic forces that have short and long-term effectiveness [51]. The diathermic effects increase blood flow to the target site, reduce local inflammation, increase blood flow, promote vasodilation, eliminate damaged tissue, and enhance exchange of nutrients [55]. The ultrasound mechanotransduction process actives the transmembrane ionic channels and regulate the cellular metabolism [56–58]. The intracellular FAK/NF-κB/P13K/MAPK pathways are also activated with stimulation leading to cellular proliferation, migration [59–61]. Collectively the long-duration ultrasound treatment provided by SAM pass deep into the tissue, increasing vessel diameter and blood flow at the injury site (Fig. 1C). The acoustic force increases the permeability of capillary epithelial walls and matrix, allowing the release of nutrients and removing cytokines and damaged tissue (Fig. 1B). Long-term application of SAM augments the healing process by increasing cellular proliferation rate (Fig. 1A).

This systematic review and meta-analysis aim to summarize the clinical effects of SAM treatment on musculoskeletal injuries including diathermy (tissue heating), functional outcomes (strength and range of motion), quality of life, pain reduction, and safety profile of the intervention.

## Methods

### Protocol

This systematic review and meta-analysis were performed and reported in accordance with the guidelines described by The PRISMA 2020 statement [62].

### Inclusion criteria

Studies were included if they applied SAM treatment to human participants (aged 18 and over) with institutional review board approval or exemption; and if they were published in English, original research or peer-reviewed, related to the musculoskeletal treatment (musculoskeletal injuries, musculoskeletal pain, pre or post operative rehabilitation, mechanistic biological stimulation, or human-factor usability); level IV (case cohort) or higher evidence based on Levels of Evidence, Oxford Centre for Evidence-Based Medicine, 2009; used validated outcome measurement methods (musculoskeletal pain, musculoskeletal function, musculoskeletal biological measures, musculoskeletal heating, therapeutic complications and/or adverse events); included study designs of comparative, case cohort or qualitative studies.

### Search strategy

**Fig. 2:**
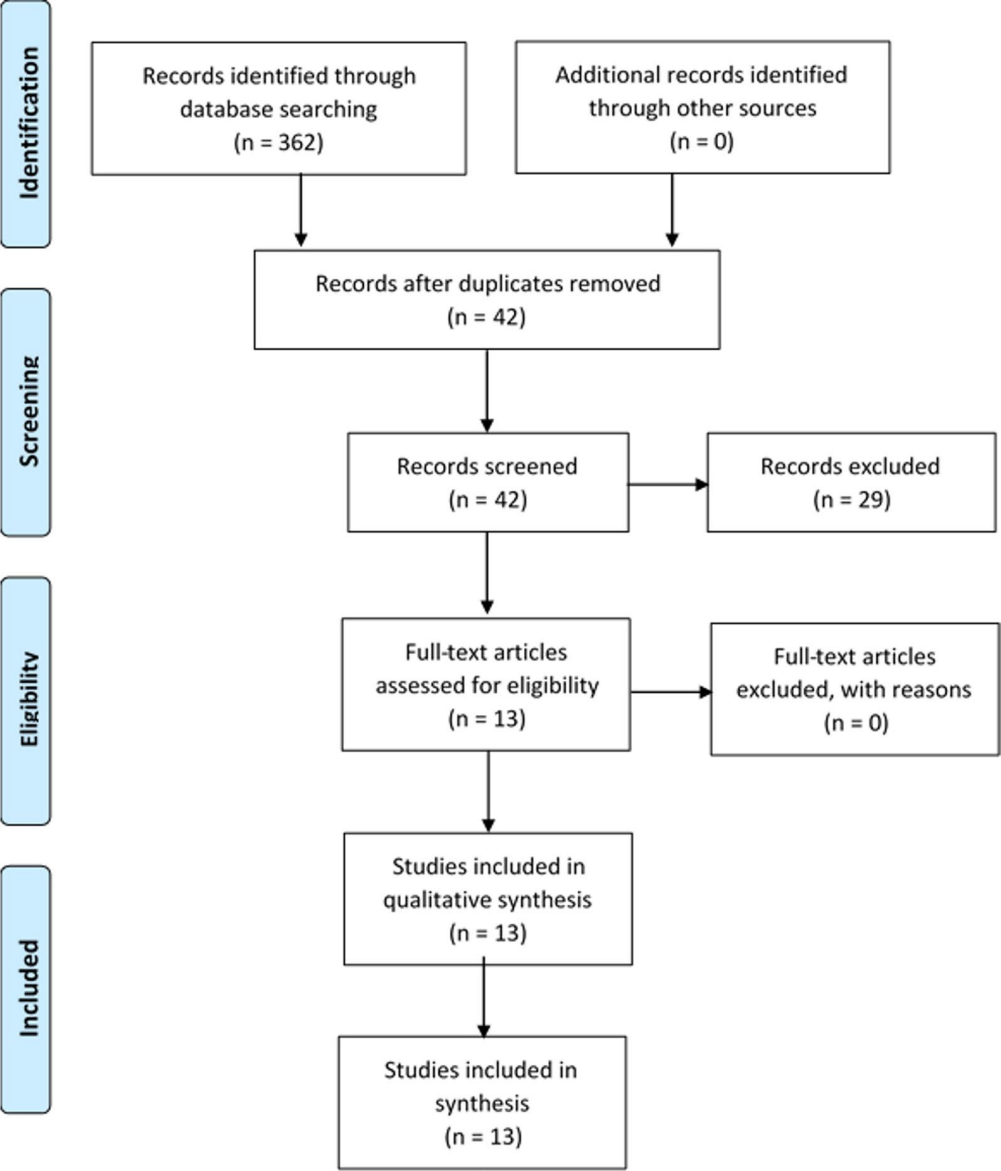
PRISMA flow diagram for identification, screening, eligibility and included articles in SAM clinical study analysis

Relevant literature was searched to identify studies of level IV or higher (Oxford Centre) measuring clinical benefit of the SAM device in clinical research applications up to 09/10/2021. Measurable clinical outcomes included: pain, function, tissue-heating (diathermy), strength, recovery, and return to work. The PRISMA flow diagram for identifying relevant research is shown in Fig. 2. PubMed, EBSCOhost, Academic Search Complete, Google Scholar and ClinicalTrials.gov search engines and databases were queried with the search terms used for identifying studies: “Sustained Acoustic Medicine” OR “SAM” (n = 62), “Low-Intensity Therapeutic Ultrasound” OR “LITUS” (n = 160), “Low-Intensity Continuous Ultrasound” OR “LICUS” (n = 120), “Wearable Therapeutic Ultrasound” (n = 20), “Low-Intensity Wearable Ultrasound” (n = 7). The search was limited to 2011–2021, i.e., the last ten years. Combination of search terms with “AND” and “OR”, along with a review of references cited within identified studies and related articles was used to uncover all relevant literature on Sustained Acoustic Medicine treatment.

### Study selection

All references were exported, and duplicates removed. Two investigators (SW, TB) screened titles and abstracts as per the inclusion criteria and retrieved full text for further analysis. Disagreements were resolved by a third reviewer (AU).

### Data collection process

The two investigators (SW and TB) independently extracted data from the selected studies and complied them into tables. The data collected included study characteristics (authors, date of publication, study design and clinical registration), study musculoskeletal focus area (body location, injury type, condition treated), and variables associated with measurable outcomes (pain, function, quality of life, diathermy, return-to-work, adverse events, safety profile and participant satisfaction). Extractable variables were pooled and stratified to similar conditions on reported outcomes in the literature for synthesis.

### Risk of bias and quality of evidence assessment

A total of 362 records were identified and a total of n = 13 clinical studies including five (5) level I, four (4) level II and four (4) level IV studies were selected for assessment. The quality of each selected study was scored by two investigators (SW and TB) using the Downs and Black checklist [63] and investigator (AU) was consulted in the cases of discrepancy. The Downs and Black 27 question check list has a maximum score of 28 points and provides detailed quality evaluation of randomized controlled and non-controlled studies for external validity, internal validity, and power. Downs and Black quality scores were tabulated for all studies based on the following tiers (poor quality < 14, fair quality 14–18, good quality 19–23 and excellent quality > 23).

### Synthesis of results

Two authors (SW and TB) completed the analysis using both Microsoft Excel (Microsoft, USA) and Review Manager Version 5.4 (The Cochrane Collaboration, Denmark). A fixed effects meta-analysis with standardized mean difference (SMD) statistics was used to analyze the results where two or more controlled studies could be analyzed. The I^2^ statistic was used to assess study heterogeneity within the meta-analysis. Analysis was conducted on studies grouped by body location and condition being treated, and according to outcomes measure (pain, health improvement and tissue heating). Given the limited evidence uncovered (13 studies) and variability in design amongst the studies (joint, tendon and soft tissue pain; function elbow, knee, and ankle; soft-tissue health improvement, deep heating of muscle tissues, biological measures of lactic acid), data groupings were made according to time points analyzed and reported and standardized for comparison controls where available. In cases where it was not possible to undertake meta-analysis such as limited evidence for a specified comparison (lack of a control group) and/or only one study available on an outcome, data was extracted into tables and main findings reported.

## Results

### Study selection

The PRISMA flow diagram is shown in Fig. 2. The search strategy yielded a total of 362 citations from the four search engines and clinical trial databases. No additional included studies were retrieved from other sources including references lists, related articles, manual searching or Cochrane library and EMBASE databases. After removing the 320 duplicates uncovered, 42 citations were screened by reading the study abstract. The remaining 13 relevant records were then analyzed for eligibility based on full text availability and inclusion criteria. The thirteen (13) clinical studies meeting inclusion criteria were divided as follows: upper shoulder, neck and back (Table 1), knee joint (Table 2), and soft tissue injuries of the musculoskeletal system (Table 3).

**Table 1:** The clinical studies conducted evaluating SAM in upper shoulder, neck and back pain in different demographics

Reference	Lewis et al. [53]	Lewis et al. [65]	Petterson et al. [64]
Study design	Randomized double-blind study	Non-randomized study	Randomized double-blinded study
Demographics	Sample size (n = 30), active (n = 20, 10 males, 10 females), placebo (n = 10, 9 male, 1 female)	Sample size (n = 5), gender not available	Sample size (n = 33), active (n = 25, 9 males, 16 females), placebo (n = 8, 3 males, 5 females)
Clinical criteria	**Trapezius Myofascial Pain** **Inclusion criteria:** age 40–60 years, physician clearance, patient consent, physician clearance, unable to self-administered treatment daily, able to record changes in pain score (VAS: 40–70) **Exclusion criteria:** Neuropathy, psychologically unstable, pregnancy, prisoner, surgery within last 6 months, no use of topical agents during and past 30 days of treatment, surgery or injury, other severe pain, self-evaluation of the trapezius myalgia	**Rotator Cuff Tendinopathy** **Inclusion criteria:** age > 40, Physician’s diagnosed rotator cuff pain (tendinosis, tear, tendonitis, adhesive capsulitis, biceps tenosynovitis, proximal humerus fracture), limited shoulder mobility, taking pain medication at least one week before treatment **Exclusion criteria:** Metastatic or infectious shoulder pathology, cervical radiculopathy, unable to utilize the device, cognitively impaired	***Upper Shoulder and Neck Pain*** **Inclusion criteria**: age 30–36 years, diagnosed for upper trapezius trigger point by a health practitioner or trainer, NRS ≥ 3 out of 10, restricted mobility **Exclusion criteria:** Neuropathy, type I/II diabetes, surgery within last 6 months at the treatment site, skin irritation to ultrasound gel. Instructed to stop other topical analgesics, discontinue other pain medication if possible
Methodology	SAM therapy for at least 1-h treatment at the onset of trapezius spasm, all the treatments were conducted at home, patient convenience, VAS metrics were recorded daily by patients, and GROC was recorded at the end of the study. At least 10 treatments with 100% compliance	SAM therapy daily, 4 h, over 12 treatments	SAM therapy over 4 h. The double-blinded and random distribution was conducted based on baseline NRS and GROC. Patients recorded changes in pain in a daily dairy
Outcomes	VAS (0–100 mm scale) GROC (over 10 days) (pre and post each treatment: − 7 to 7) Pain reduction post-treatment (120 min treatment)	VAS (1–100 mm scale) GROC (pre and post per treatment: − 7 to 7)	NRS: (1–10 scale) GROC: (pre and post per treatment: − 7 to 7)
Main findings	VAS: The most pain reduction was in the first 2 days (active mean 21.25% ± 9%, placebo mean 4% ± 9%, *p* < 0.05), over 10 treatment (active mean 16% ± 7.5% vs. placebo mean 7.5% ± 7.5%, *p* < 0.05) GROC: 60% improvement relative to placebo over 10 days treatments. Males were more responsive to treatment than females (*p* < 0.05) Post-treatment pain reduction in males was 78% and females 52% over the first hour (*p* < 0.05)	VAS: 30% improvement over 12 sessions on scale (*p* < 0.05) VAS: 52% improvement over starting VAS score (*p* < 0.05) GROC: 52% improvement over 12 sessions (*p* < 0.05)	NRS: Over 4 weeks of treatment, patients reported 2.61 points to decrease (*p* < 0.001). pain decrease of 1.03 points over placebo treatment (p = 0.003) GROC: 4-week treatment improvement by 2.84 points in the treated group relative to 0.46 in the placebo (*p* < 0.001)
Level of evidence	1C	4	1B
Downs and black score	21/28, good quality	11/28, poor quality	26/28, excellent quality
Conclusion	Clinical study reported the SAM efficacy in chronic trapezius myofascial pain. The primary outcomes VAS, GROC, and pain reduction post-treatment recommends further clinical studies. No adverse side effects	Preliminarily study reporting an increase in shoulder mobility, reduction in pain and improved quality of care with no placebo control	SAM treatment has clinically significant outcomes to reduce pain and improve quality of life at the study dosing protocol

Significance was defined as a probability value less than 0.05

Measurable outcomes include pain reduction and health improvement

Numeric Rate of Pain Scale (NRS, 1–10), Visual Analogy Scale (VAS, 1-100 mm), Global Rate of Change Scale (GROC, -7 to + 7)

**Table 2:** The clinical studies of SAM efficacy on knee osteoarthritis symptoms

Reference	Langer et al. [67]	Langer et al. [51]	Draper et al. [52]	Madzia et al. [66]
Study design	Clinical study	Randomized, placebo-controlled clinical study	Double-blind randomized placebo-controlled clinical study	Multi-site clinical efficacy study
Demographics	Study 1: sample size (n = 12, no placebo), VAS pain focused Study 2: sample size (n = 7, 4 active, 3 placebo), Pain and mobility focused	Sample size (n = 47, active (n = 28), placebo (n = 19)	Sample size (n = 90, 23 males, 28 females), active (n = 55), placebo (n = 35, 16 males, 17 females)	Total sample size (n = 32, 18-males, 14- females), Rapid responders (n = 24)
Clinical criteria	**Knee osteoarthritis** **Inclusion criteria:** Diagnosed with mild to moderate knee OA, between 35–80 years, reported a frequent pain score of 3 to 7 on the VAS during the week preceding enrollment, deemed appropriate to participate by the study physician **Exclusion criteria:** Not defined	**Knee osteoarthritis** **Inclusion criteria:** Radiographic mild to moderate clinical knee osteoarthritis (Grade 1–2 on the OARSI scale) in one or both knees, average pain score > 4 on a 10 point (0-100 mm) VAS scale during the week prior to enrollment **Exclusion criteria:** Not defined	**Knee osteoarthritis** **Inclusion criteria:** Age (35–80 years), mild, moderate OA K/L grade I/II, OA in one or both knees, Osteophytes, joint space, NRS between 3 and 7, self-apply device **Exclusion criteria:** Severe OA (K/L III), knee replacement (TKA), surgical intervention, hyaluronic acid injection in the last 6 months, non-ambulatory patient, no corticosteroids, osteoarthritis due to secondary metabolic disorder	**Knee osteoarthritis** **Inclusion criteria:** Age 45–85, K/L II – III grade, NRS between 3 and 7 (0–10 scale) **Exclusion criteria:** Patients with no intra-articular injection in the last 6 months, no trauma, no implants or surgeries at the arthritic knee, K/L greater than III, steroid base medication, OA due to other metabolic disorders **Rapid responders:** Patients reporting 1-point NRS pain reduction after the first treatment (n = 24)
Methodology	SAM therapy for 4–8 h daily VAS: 12 – 60 days, daily pain diary was maintained by patients at home Mobility: 6 weeks, data recorded at 2 weeks increments using actigraph. Patients also recorded pain in the morning, afternoon, and evening	SAM therapy for 4 h per day at least four times per week for six (6) weeks, recording their pain before and after treatment in dairy. Participants attended bi-weekly visits to the clinical study site to assess compliance	SAM therapy over 6 weeks, self-administered in the home setting for 4 h per day Patients recorded NRS post-treatment, and WOMAC score was recorded after 6 weeks Range of motion and strength testing using manual muscle tester at approximately 90 degrees	SAM therapy NRS was recorded by patients daily pre-and post-treatment by the patient for 7 days WOMAC score was recorded and start and end of study at the outpatient center
Outcomes	VAS: (0–100 mm) Mobility: actigraphy	VAS: (0–100 mm)	NRS (0–10 Scale) WOMAC (0–960, pain, stiffness, functionality) Range of motion and strength (n = 17 sub cohort)	NRS: (0–10 pain scale) WOMAC (0–960, pain, stiffness, functionality)
Main findings	VAS pain decreased by 52% over 60 days (*p* < 0.05) 20% improvement in mobility over 6 weeks	VAS pain decrease by 2.5 points (1.23 over placebo, *p* < 0.03) for subjects with moderate to severe starting pain	1.96 point NRS pain relative to 0.85 placebo decrease in 6 weeks (*p* < 0.001) WOMAC: 505 points decrease in the active group relative to 311 points in the placebo group (*p* = 0.02) In pilot subset rotational strength increased from baseline to 6 weeks (3.2 N, *p* = 0.03); however, no other measures were significant	2.06 point NRS pain decrease (50%, *p* < 0.001) was reported in the entire study cohort with a 2.96 NRS point decrease in rapid responders (70%. *p* < 0.001) relative to baseline NRS pain score WOMAC scored increased by 351 in the complete cohort (*p* < 0.001) and 510 in rapid responders (*p* < 0.001) 95% of patients reported ease of use and continuation of treatment
Level of evidence	1C	1C	1B	2A
Downs and black score	17/28, fair quality	12/28, poor quality	27/28, excellent quality	23/28, good quality
Conclusion	Pain reduction in the subset of patients with no significant change in mobility. Require larger clinical study to measure mobility improvement	SAM treatment provided clinically effective pain reduction according to the Initiative on Methods, Measurements, and Pain Assessment in Clinical Trials (IMMPACT)	SAM treatment significantly decreased pain, increased mobility and rotational strength in mild to moderate OA. Further studies are required to establish effect on joint range of motion	In the efficacy study, SAM with diclofenac ultrasound gel patch showed significant effectiveness in knee OA pain alleviation and increased functionality with high usability and safety

Significance was defined as a probability value less than 0.05

The clinical evidence shows the application of SAM as standalone and adjunctive therapy for knee osteoarthritis. Early evidence suggest ultrasound treatment may play an important role in slowing down OA progression, reducing pain, and retaining patients’ mobility

Numeric Rate of Pain Scale (NRS, 0–10), Visual Analogy Scale (VAS, 0-100 mm), Western Ontario and McMaster Universities Osteoarthritis Index (WOMAC, 0–960)

**Table 3:** Soft tissue is highly heterogeneous, including skeletal muscle, smooth muscle, tendons, and ligaments

Reference	Taggart et al. [71]	Rigby et al. [55]	Best et al. [50]	Langer et al. [68]	Langer et al. [69]	Draper et al. [70]
Study design	Safety and usability clinical study	Randomized placebo controlled clinical study	Clinical case series	Randomized cross-over placebo controlled clinical study	Clinical case series	Clinical case series
Demographics	Sample size (n = 20), gender not available	Sample size (n = 26, 16 males, 10 females), active (n = 20), placebo (n = 6)	Sample size (n = 25, 11 males, 10 female), Achilles (n = 5), elbow (n = 20)	Sample size (n = 16) active (n = 16), placebo (n = 16), age 22 ± 2, (16 males)	Sample size (n = 44), normal BMI: arm (n = 11), leg (n = 11); high BMI: arm (n = 11), leg (n = 11)	Sample size (n = 18), age 30 ± 13.31, (13 males, 5 female)
Clinical criteria	**Home use of SAM treatment** **Inclusion criteria:** 18 years of age or older, with the ability to read, write, and speak English **Exclusion criteria:** Individuals were excluded if they had a condition that was contraindicated for ultrasound therapy	**Diathermic effects** **Inclusion criteria: H**ealthy subjects **Exclusion criteria:** patients with fever, lower leg infection or wound, lack of sensation. Participant were instructed not to exercise for 24 h prior to test	**Soft tissue Injury** **Inclusion criteria: A**ge 16–65 years old, no NSAIDs, pain prescription pain during the study **Exclusion criteria:** no neuropathy, no type I or II diabetes, surgery at the treatment site, malignancy, use of topical agents, application of corticosteroid or platelet-rich plasma injection, medical or psychological condition, participate in no clinical trial in any other clinical trial for last 30 days, no trauma, open sores or wound at treatment site	**Soft tissue Injury** **Inclusion criteria:** Healthy subjects, age 20–24 years old, no NSAIDS, no massage, no nutritional supplements, previous resistance training experience **Exclusion criteria:** Not available	**Diathermic effects** **Inclusion criteria:** Healthy subjects **Exclusion criteria:** Not available	**Soft tissue injury** **Inclusion criteria:** Using adjunctive therapy, sports-related injury, and cognitively able to follow instructions **Exclusion criteria:** past surgeries, opioid-based medications, any implant, intramuscular or articular injections, and using NSAIDs
Methodology	Participants evaluated SAM device in operation mode with one and two ultrasound transducers. Subjects were asked to use the device three times within a seven day period, each time for a four-hour treatment duration. After each treatment, subjects were asked to complete a 27-question quiz that assessed how the device was used, where it was applied, the ease of use, whether the device was operated successfully, and a discussion of any issues that may have been encountered while wearing the device	Participants were treated with one or two transducers. Two transducers were placed 8.5 cm apart at room temperature, change in temperature was recorded at 1.5 cm and 3 cm intramuscular depth using thermocouplers (MT 23/5; Physitemp Instruments LLC, Clifton, NJ). Change in intramuscular temperature was continuously recorded for 3 h. The study was conducted at room temperature	Injury sites were treated with SAM therapy, 4-h daily for 6 weeks. Subject self-reported pain per day during treatment at 30 min, 2 h, and the end of treatment. A dynamometer was used to measure force generation and grip strength daily for elbow pathology	SAM therapy applied to the quadriceps and hamstrings for 1 h prior to exercise, used throughout exercise and recovery after exercising up to 4 h of SAM treatment. Exercise included two sets at 70% maximum of lunges, seated hamstring flexion, smith squats, seated quadriceps extension; and leg press. Blood-lactate measured at baseline and through workout and recovery periods. Muscle performance measured (total work, peak torque, and average power) in the dominant leg post exercise	Participants stratified into normal and high BMI. Applied SAM therapy on the arm or leg with two ultrasound transducers. On the arm SAM was applied to the elbow and forearm. On the leg, SAM was applied to the knee and calf. A temperature measurement thermocouple was placed between the SAM device and skin to measure temperature over 4 h of treatment. Participants were asked to remain still during measurement to prevent excessive movement from disturbing thermocouple placement	Athletes were treated with SAM over 4 h of adjunctive therapy. Duration of therapy was determined as required by physical therapy staff
Outcomes	Device ease of use Subject overall experience Adverse events from use	Diathermic changes during SAM treatment	NRS pain (1–10 scale) Grip strength (N) and force generation (N)	Blood lactate levels (mmol·min·L^−1^) Ave Power (W) Total work (N-m) Peak Torque (N-m)	Diathermic changes during SAM treatment	NRS pain (1–10 scale) Quality of life Return to sport
Main findings	95% of subjects were able to successfully operate the device 93% of subjects thought the device was easy to use 90% of subjects had a positive experience overall 87% of subjects would use the device again No adverse events reported from treatment	1 transducer treatment showed 4.45C increase at 1.5 cm and 3.18C at 3 cm over placebo 2 transducers increased intramuscular temperature by 3.95C at 1.5 cm and 3.22C at 3 cm over placebo	3.94 point NRS decrease was reported in elbow tendinopathy (*p* = 0.002) 2.83 kg improvement in grip strength improvement (*p* = 0.04) Among 5 subjects with Achilles tendinopathy, a reduction in pain and improvement in strength was observed	Reduction of blood lactate by 20%, (255.8 ± 120.0 mmol min L^−1^) versus placebo condition (318.5 ± 86.0 mmol min L^−1^) *p* = 0.002 Increased average power (*p* = 0.024) Increased work (*p* = 0.031) Increased torque (*p* = 0.031)	Elbow: 12C temperature increase Forearm 12C temperature increase Knee 12C temperature increase Calf: 13C temperature increase	Athletes reported on average 3.33 point decrease in NRS pain score (*p* < 0.05) 87% improvement in function/quality of life, and 55% of were able to return to the sporting activity post-treatment
Level of evidence	4	2B	4	2B	4	4
Downs and black score	19/28, good quality	21/28, good quality	21/28, good quality	25/28, excellent quality	16/28, fair quality	15/28, fair quality
Conclusion	SAM treatment holds the promise of providing non-pharmaceutical pain relief to patients suffering from a broad range of conditions. The device was successful in providing home ultrasound treatment, and 9:10 subjects who tried the device would use it again	SAM treatment increased the intramuscular temperature by 3°–4°. Further studies are required to assess physiological changes	SAM treatment shows potential as an effective treatment for elbow and Achilles’ tendinopathy. No adverse effects were reported	The use of SAM after exercised induced muscle damage can reduce lactic acid and improve some measurements of muscle performance in the lower extremities	SAM treatment provides over + 12C of diathermy while maintaining skin temperature at a maximum of 40C. SAM is a viable and safe treatment to delivering the biophysical effects of ultrasound	SAM therapy as adjunct therapy can expedite the rehabilitation process in musculoskeletal injuries

Significance was defined as a probability value less than 0.05

Six clinical studies have reported on the effectiveness and ease of use of SAM therapy. The continuous vigorous deep diathermic effects of SAM therapy is believed to be one mechanism to reduce pain, accelerate healing and recovery of soft tissue injuries as observed in studies (Best et al. 2015, Langer et al. 2017 and Draper et al. 2020)

Numeric Rate of Pain Scale (NRS, 0–10)

### Upper neck, back and shoulder conditions

#### Study characteristics and participants

The study characteristics and participants for upper neck, back and shoulder conditions are reported in Table 1. The three eligible studies comprised two randomized controlled trials (RCTs) [53, 64] and one prospective non-randomized study [65]. Two of the three studies compared an intervention group (SAM) with a placebo control group (Non-Functioning Device) in the treatment of upper back myofascial pain and upper shoulder and neck pain, and one study evaluate SAM in a case cohort on rotator cuff tendinopathy. Among the included studies, two were single center conducted in the United States [53, 65]. One was a multicenter trial conducted in the United States [64]. The included studies involved a total of 67 participants who received SAM treatment in additional to usual care for musculoskeletal injury or pain. One study included patients 40–60 years of age with chronic trapezius myofascial pain [53], one study included younger 30–36 years of age patients with episodic upper shoulder and neck pain [64], and another study on shoulder tendinopathy included patients over 40 years of age [65]. Both men and women were equally represented in the include studies (31 males, 32 females, 5 unreported).

#### Study intervention characteristics

The characteristics and methodology of SAM treatment for upper neck back and shoulder conditions are reported in Table 1. Two studies applied SAM treatment with one ultrasound delivery head operating at 2.5–3 MHz, 0.44–0.65 W and 89.6–90 mW/cm^2^ for 1–4 h, respectively [53, 65]. One study applied two SAM ultrasound delivery heads operating at 3 MHz, 1.3 W (0.65 W each), 132 mW/cm^2^ for 4 h [64]. SAM treatment was applied during heightened or breakthrough pain in two studies [53, 64], and on a daily treatment regimen for shoulder injury in the other study [65]. All three studies used SAM for at least 10 treatment sessions over a course of two weeks, and one study applied the intervention for 4 weeks [64].

#### Level of evidence and quality of studies

The level of evidence and quality assessment of the studies is shown in Table 1. One study was considered poor quality [65], one study of good quality [53] and one study of excellent quality [64]. Two RCTs blinded evaluators and subjects, and clearly reported objectives, described the outcomes to be measured and the main findings [53, 64].

#### Study outcomes and main findings

The primary outcomes and main findings from the included studies are shown in Table 1. Pain reduction using the visual analog scale (VAS 1–100 mm) or numeric rating scale (NRS 0–10), and overall health improvement using the global rate of change scale (GROC: − 7 to + 7) were evaluated in all included studies and supported meta-analysis. Lewis et al. [53] in a 30 subject RCT on myofascial pain reported a 200% reduction in pain (16% vs. 7.5% *p* < 0.05) compared to placebo, and a 60% GROC improvement over the placebo group after 10 SAM treatment sessions (*p* < 0.05). In the pilot study by Lewis et al. [65] 5 patients with rotator cuff tendinopathy reported a 30% reduction in pain and a 52% improvement in the GROC after 12 SAM treatment session (*p* < 0.05). In a 33 subject RCT on upper neck and shoulder pain conducted by Petterson et al. [64] pain was reduced by 2.61 points (46.6%) for SAM treatment patients (*p* < 0.001) and a 1.03 points decrease over placebo (*p* = 0.003) after 4 weeks of intervention. Petterson et al. [64] also reported a 2.84 point GROC improvement over placebo treatment (*p* < 0.001).

**Fig. 3:**
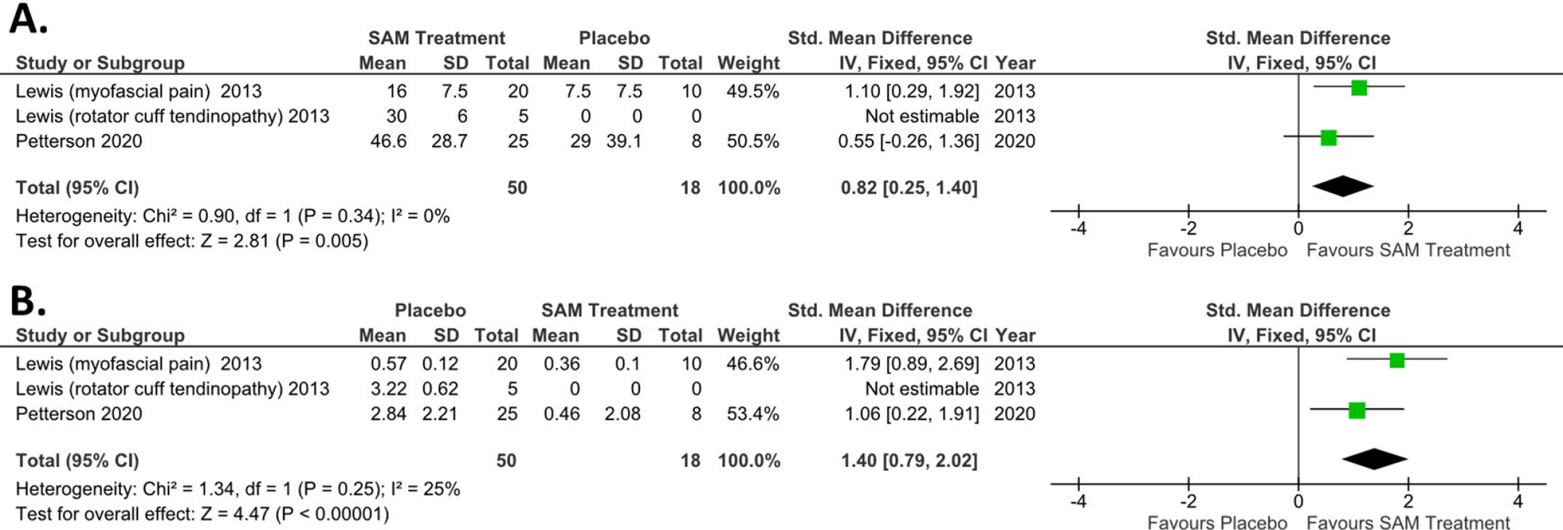
Forest plot of pain and health improvement measures comparing SAM Treatment vs. placebo **A** SAM treatment provides significant reduction of pain (*p* = 0.005). **B** SAM provides significant improvement in health quality (*p* < 0.00001)

The meta-analysis and forest plot of SAM treatment outcomes on pain and global health improvement compared to placebo treatment are shown in Fig. 3 for upper back, neck, and shoulder conditions. The availability of two randomized controlled trials provided a Pain Reduction (SMD 0.82; 95% CI 0.25–1.40; I^2^ = 0%; n = 63) and Global Health Improvement (SMD 1.40; 95% CI 0.79–2.02; I^2^ = 25%; n = 63). There were significant between-group differences found in pain (*p* = 0.005) and health improvement (*p* < 0.0001) with low heterogeneity between studies (I^2^ values ≤ 25%). The two studies in these outcomes were graded as good to excellent [53, 64].

### The knee joint

#### Study characteristics and participants

The study characteristics and participants for knee joint conditions treated by SAM are reported in Table 2. The four eligible studies comprised two randomized controlled trials (RCTs) [51, 52] one prospective multi-site non-randomized study [66] and two combined pilot studies [67]. Three of the four studies compared an intervention group (SAM) with a placebo control group (Non-Functioning Device) in the treatment of mild to moderate grade knee Osteoarthritis on clinically validated scales (Kellgren and Lawrence system for classification of osteoarthritis or Osteoarthritis Research Society International Scale). Among the included studies, three were single center [51, 52, 67] and one was a multicenter trial [66] all conducted in the United States. The included studies involved a total of 156 subjects who received SAM treatment in additional to usual care for knee joint pain. All studies included patients 35–85 years of age with chronic knee Osteoarthritis pain and radiographic diagnosis. Men represented 57% and women 43% of the described study populations across the four studies (41 males, 31 females, 66 unreported).

#### Study intervention characteristics

The characteristics and methodology of SAM treatment for knee joint pain related to Osteoarthritis is shown in Table 2. All four studies applied SAM treatment at 3 MHz, 132 mW/cm^2^ for 4 h daily [51, 52, 66, 67]. One study applied one SAM ultrasound delivery head operating delivering 0.65 W of energy [67], the other three studies applied SAM treatment with two ultrasound delivery heads operating at 1.3 W over for 4 h [51, 52, 66]. One study utilized a 1% diclofenac ultrasound coupling gel with the intervention [66]. SAM treatment was applied to the knee daily with patients reporting baseline pain scores from 3 to 7 on the 10-point scale. Three studies applied SAM for at least 6 weeks of treatment [51, 52, 67] and one study applied SAM for one week of treatment [66].

#### Level of evidence and quality of studies

The level of evidence and quality assessment of the studies is shown in Table 2. One study was poor quality [51], one study was fair quality [67], one study was good quality [66] and one study of excellent quality [52]. Two RCTs blinded evaluators and subjects, and clearly reported objectives, described the outcomes to be measured and the main findings [51, 52]. Two studies lacked detail and were preliminary pilot studies or short reports on registered studies [51, 67].

#### Study outcomes and main findings

The primary outcomes and main findings from the included knee joint studies are shown in Table 2. Pain reduction using the visual analog scale (VAS 1–100 mm) or numeric rating scale (NRS 0–10) was used in all four studies. Knee joint functional improvement using The Western Ontario and McMaster Universities Arthritis Index (WOMAC: 0-960 scale including 24 questions related to pain, stiffness, and function) score was applied across two studies [52, 66], and range of joint motion was evaluated in one study [52]. The study conducted by Langer et al. 2014 showed the initial usability of SAM treatment for knee OA [67]. Patients, on average, reported a 52% reduction in the pain score from baseline with no adverse effects and 95% satisfaction of treatment (p < 0.05). Langer et al. 2015 in a clinical review on SAM, reported results of a 47 subject randomized placebo-controlled study evaluating treatment on Knee Osteoarthritis [51]. Over 6 weeks, SAM reduced pain on the VAS by 2.5 points which was statistically different from the 1.23-point decrease of the placebo group (*p* < 0.03). A 90-subject double-blinded, placebo-controlled study by Draper et al. 2018 reported a 1.96-point decrease in NRS pain relative to placebo treatment (*p* < 0.01) [52]. WOMAC function, stiffness and pain score improved by 505 points for the SAM treatment group (*p* < 0.01). In a 32 patient multicenter study on knee Osteoarthritis, Madzia et al. [66] reported 2.06 -point 50% pain decrease in the entire cohort (*p* < 0.001) and 2.96-point 75% pain decrease in responders (*p* < 0.001). The WOMAC score improved by 351 points in the entire population (*p* < 0.001) and 510 points (*p* < 0.001) in the rapid responder cohort. A high usability rate over 95% patient satisfaction and no adverse events were also reported.

**Fig. 4:**
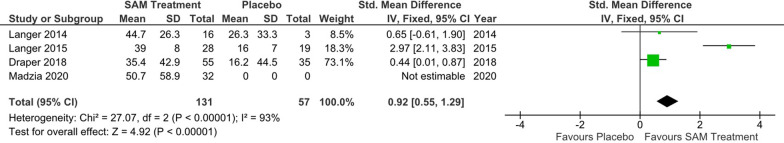
Forest plot of knee Osteoarthritis pain reduction from SAM Treatment vs. placebo. SAM treatment provides significant reduction of pain (*p* < 0.00001)

The meta-analysis and forest plot of SAM treatment outcomes on pain reduction compared to placebo treatment are shown in Fig. 4 for the knee joint. The availability of two randomized controlled trials and one controlled pilot study provided a Pain Reduction (SMD 0.92; 95% CI 0.55–1.29; I^2^ = 93%; n = 63). There were significant between-group differences found in pain (*p* < 0.00001). The included studies had high heterogeneity (I^2^ values ≥ 50%) which was not meaningfully reduced by exclusion of a data set. The three studies included in these outcomes were graded as poor to excellent quality. Other measures such as WOMAC were not sufficiently available to conduct analysis on.

### Patient-self treatment and soft tissue injuries

#### Study characteristics and participants

The study characteristics and participants for patient-self-treatment and soft tissue injuries treated by SAM are reported in Table 3. The six eligible studies comprised two randomized controlled trials (RCTs) [55, 68], three clinical case series [50, 69, 70] and one safety and usability study [71]. Two of the six studies compared an intervention group (SAM) with a placebo control group (Non-Functioning Device) in proving deep heat (ultrasonic diathermy) to muscle tissue and increasing muscle performance and recovery after high impact exercise [55, 68]. All six studies were single center trials conducted in the United States. The included studies involved a total of 149 subjects who received SAM treatment in a variety of musculoskeletal injuries and/or were conducted to measure SAM therapeutic performance and mechanisms of action in human subjects. Two studies were conducted on deep tissue heating on various locations of the body [55, 69], one study on quad and hamstring muscle performance and biomolecular measures [68], one human-factor usability and safety study applying SAM to various physical locations on the body [71], and two clinical studies focused on healing soft-tissue injuries to musculoskeletal tissue [55, 70]. Studies included both injured and healthy subjects 18 years of age or older, men represented 69% and women 31% of the described study populations across the four studies (56 males, 25 females, 64 unreported).

#### Study intervention characteristics

The characteristics and methodology of SAM treatment for patient self-treatment and soft tissue injuries is shown in Table 3. All six studies applied SAM treatment at 3 MHz, 132 mW/cm^2^ for 4 h daily [50, 55, 68–71]. Two studies varied SAM treatment between one and two ultrasound delivery heads operating delivering 0.65 W or 1.3 W to determine usability and safety [71] and deep heating (diathermy performance) [55]. The other four studies applied SAM treatment with two ultrasound delivery heads operating at 1.3 W over for 4 h [55, 68–70]. One study utilized four SAM devices on each subject during regular therapy sessions delivering 5.2 W [68]. Across all six studies SAM treatment was applied directly over the injury site or over the specified target region looking to be evaluated.

#### Level of evidence and quality of studies

The level of evidence and quality assessment of the studies is shown in Table 3. Two studies were fair quality [69, 70], three studies were good quality [50, 55, 71] and one study of excellent quality [68]. Two RCTs blinded subjects, and clearly reported objectives, described the outcomes to be measured and the main findings [55, 68]. One prospective case series attempted to blind subjects of the treatment [50]. The remaining three studies included sufficient detail but did not have specific controls due to the study design and/or purpose [69–71].

#### Study outcomes and main findings

The primary outcomes and main findings from the included studies on self-treatment and soft tissue injury are shown in Table 3. Soft tissue injury pain reduction using numeric rating scale (NRS 0–10) was used in two studies [50, 70]. Measures of functional improvement such as range of motion, dynamometer, strength, and power were applied in three studies [50, 69, 70]. Two study measured diathermy temporal heating profiles with thermocouples in situ [55, 69]. One study included blood measures of lactic acid clearance [68]. One study included usability and satisfaction of treatment [71].

A placebo-controlled study by Rigby et al. 2015 (n = 26 subjects, 20 active, 6 placebo) measured the diathermic effects of one and two SAM transducer setups at 1.5 cm and 3 cm intramuscular depth over 3 h [55]. The 3–4 °C temperature increase occurred over 3 h, leading to increased blood flow, vasodilation, and oxygenation of the intramuscular tissue. Langer et al. 2017 (n = 44 subjects, 22 normal body mass index (BMI) and 22 high BMI) evaluated two SAM transducers for diathermy on the elbow, forearm, knee, and calf [69]. Langer et al. 2017 compared clinical experimental data to mathematical modeling of the diathermy generated by SAM [69]. Over the 4-h SAM treatment and temperature recording, the temperature directly below the SAM ultrasound transducer increased from 12 to 13 °C in approximately 20 min of use and sustained for the duration of treatment. The prediction model of diathermy was able to predict the clinical measurements closely. A human-factor clinical usability study by Taggart et al. 2014 (n = 20 subjects) evaluated the effective application of SAM treatment in the home and clinic setting [71]. Over 60 unique SAM treatment sessions, 95% of subjects successfully applied and operated the device, and 93% found the treatment easy to use.

Best et al. 2015 reported the efficacy of SAM therapy in controlled case studies including Achilles and elbow tendinopathy (n = 25 subjects) [50]. Patients were treated for 4 h. daily over 6 weeks. Patients reported change in pain at 30 min, 2 h, and the end of each treatment (4 h). Dynamometer force and grip strength measurements were taken pre and post. Patients reported a 3.94 point on average reduction in pain over 6 weeks on NRS pain scale (*p* = 0.002) and a 2.38 kg improvement in grip strength (*p* = 0.04). An overall reduction in pain was observed within the 4-h treatment sessions (*p* < 0.001) as well.

A randomized placebo-controlled cross-over study design by Langer et al. 2017 reported on the efficacy of SAM treatment to improve healing and recovery after muscle injury from high-intensity resistant exercise (n = 16 subjects) [68]. Subjects completed a series of five lower-body resistance exercises with active and placebo SAM treatment applied to the quadriceps and hamstrings at rest and during the exercise bouts. Blood lactate concentration was measured along with isokinetic dynamometer measurements during leg extension and flexion exercises. At each post-exercise time point measured, the lactate concentration was reduced in the active treatment 255.8 ± 120.0 mmol min L^−1^ compared to the sham treatment 318.5 ± 86.0 mmol min L^−1^ (*p* = 0.002), reflecting a 20% average decrease in total blood lactate levels after 1 h of recovery with SAM. There were also improvements in muscle performance with active versus placebo SAM treatment, including increased peak torque at 90° sec^−1^ into extension (*p* = 0.031), increased total work at 90° sec^−1^ into extension (*p* = 0.027) and average power output at 90° sec^−1^ into extension (*p* = 0.024). Similarly, Draper et al. 2020 conducted a set of case studies (n = 18) using SAM as an adjunct therapy in athletic injuries from sports [70]. The therapy was applied at various anatomic sites targeting multiple soft tissues such as ligament, muscle, and tendon. The study reported NRS, quality of life, and return of return to sports as outcome measures. There was a 3.33-point decrease in NRS pain score (*p* < 0.05), 87% improvement in quality of life, and 55% of the athletes successfully returned to active sports.

**Fig. 5:**
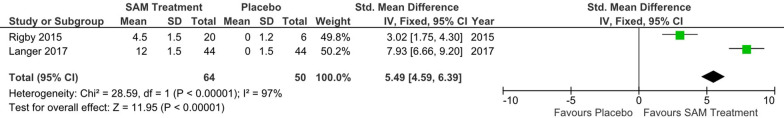
Forest plot of ultrasound diathermy tissue heating with SAM Treatment vs. placebo. SAM treatment provides significant temperature increase of soft-tissue (*p* < 0.00001)

The meta-analysis and forest plot of SAM treatment outcomes on diathermy compared to placebo/no-treatment are shown in Fig. 5. The availability of one randomized controlled trial and one case series with baseline measure provided demonstrated increased heating (SMD 5.49; 95% CI 4.59–6.39; I^2^ = 97%; n = 114). There were significant between-group differences found in tissue heating (*p* < 0.00001). The included studies had high heterogeneity (I^2^ values ≥ 50%) related to location (internal vs. external) diathermy measurement on the body. The two studies included in this outcome were graded as fair to good quality. Other measures such as pain, lactic acid, and functional measures were not sufficiently available to conduct meta-analysis on.

## Discussion

Musculoskeletal pain and soft-tissue injuries are highly prevalent with a significant impact on quality of life and the economy [5]. Acute pain is treatable with standard short-term use of NSAIDs, but chronic pain can significantly impair daily life. The transition from acute to chronic pain has been an unmet challenge in clinical sciences [14]. Pain management has been an ongoing research topic, but there is a need for therapies that are not limited to pain management and expedite the healing process by activating underlying physiological processes at the tissue, cellular, and molecular level. Current strategies employing NSAIDs, and opioid-based drugs have well known healing limitations and risks [2, 46, 72]. The overuse of NSAIDs has a significant adverse effect on gastric organs, kidneys, and liver, while overuse of opioid-based drugs has led to the opioid pandemic [2]. Neither of these therapies has regenerative effects; they ultimately lead to invasive procedures such as total hip arthroplasty, total knee arthroplasty, rotator cuff surgeries, etc. [73, 74].

As a recently approved FDA home-use treatment in 2020 [49], this systematic review and meta-analysis aimed to investigate and summarize the effects of Sustained Acoustic Medicine (SAM) therapy in musculoskeletal applications for the healthcare community. The clinical literature on SAM demonstrates it as a clinically effective mechanobiological that applies low-intensity continuous high-frequency ultrasound at 3 MHz, 132 mW/cm^2^ and delivers 18,720 J of energy over 4 h of treatment [50, 55, 65, 75]. It is a prescribed, in-home treatment, that requires little or no supervision from medical staff. Long-duration treatment with SAM shows little or no adverse effects [75]. The treatment is unique as it applies both mechanical and thermal stimuli to activate various cellular and molecular pathways for active pain management and regeneration of damaged tissue. SAM inhibits inflammation, slows down the degeneration, promotes migration of cells, and induces regeneration of new tissue. Collectively, SAM therapy helps in pain management and regenerate mechanically and physiologically functional tissue. SAM is a candidate treatment to manage soft tissue pain and amplify the healing of soft tissue injuries [64, 65].

The studies conducted by Lewis et al. 2013 and Petterson et al. 2020 show SAM’s ability to manage pain in fibrous and skeletal tissue in the upper shoulder and neck, alleviate pain, and increase shoulder mobility [53, 64]. In addition, studies conducted by Langer et al. 2014, 2015 and Draper et al. 2018 show the efficacy of SAM as a standalone therapy in mild to moderate knee OA [51, 52, 67]. Meta-analysis of the primary outcomes for the pooled studies favored SAM treatment over control, and provides evidence of effective use of SAM and the convenience of home use.

Case series by Best et al. 2015 and Draper et al. 2020 reported the effects of SAM treatment on fibrous and skeletal tissue [50, 70]. The studies showed strong data in reducing pain, improving grip strength, and returning patients back to work. Usability and diathermic clinical studies by Taggart et al. 2014, Rigby et al. 2015 and Langer et al. 2017 demonstrated SAM as a safe and effective home-use treatment, and a treatment that provided vigorous heating to muscle tissue and various areas of the body [55, 69]. Finally, Madzia et al. 2020 showed the application of SAM as a combination therapy with diclofenac and its ability to rapidly reduce chronic joint pain by 70% or 440% greater than placebo [66].

Across the thirteen studies (n = 372 subjects) measurable outcomes on device usability, safety profile, diathermy, pain relief, health improvement, and functional assessment using dynamometry, range of motion and grip strength were measured. Table 4 presents the SAM systematic reviews pooled findings with nine (n = 9 studies demonstrating musculoskeletal pain relief), six (n = 6 studies demonstrating functional joint improvement), three (n = 3 studies demonstrating improved quality of health), three (n = 3 studies showing a mechanism of SAM biological action in situ), thirteen (n = 13 studies reporting no adverse events and excellent safety profile) and seven (n = 7 studies reporting high compliance and patient satisfaction). Both pain reduction and improved joint function have the strongest evidence for SAM in the literature with (n = 9) and (n = 6) studies, respectively reporting significant and clinically meaningful improvements. This was followed by improved quality of life and therapeutic heating reported by (n = 3) and (n = 2) studies, respectively. In sub categorical meta-analysis by body location and condition type, both pain reduction and global health score quality improvement significantly favored SAM treatment. Of the thirteen (n = 13) studies reported herein, 7 of 13 were registered on the national clinical trials database (Table 4). Cumulatively, these studies demonstrate the efficacy of SAM therapy as standalone or adjunctive therapy for the upper back, neck, shoulder, knee, and soft tissue pain reduction along with improved patient mobility, functionality, and return to regular day-to-day life after an injury. The data presented in these clinical studies show positive and significant benefit for patients. Furthermore, recent health economic and SAM practitioner survey analysis support medical guideline adoption for SAM as a novel mechanobiological treatment for patient care [77, 78]. SAM treatment which is widely used in sports medicine, may be considered more broadly as a noninvasive, safe, and effective treatment option for patients with musculoskeletal pain and soft-tissue injuries [78].

**Table 4:** Summary of measurable outcomes on pain, function, quality of life and compliance for SAM clinical studies in the peer-reviewed literature

Author	Pain reduction VAS/NRS	Improved function (WOMAC/ROM/dynamometry)	Improved health (GROC)	Mechanistic outcomes included	Clinical trail registration number	Safety profile	Subject receptivity and compliance to treatment
Lewis et al. [53]	YES: 16–21% *p* < 0.05, *p* = 0.03	Not measured	YES: 60% *p* = 0.05	NA	NA	Excellent	YES: 100% compliance to treatment
Lewis et al. [65]	YES: 30–52% *p* < 0.05	Not measured	YES: 52% *p* < 0.05	NA	NA	Excellent	NA
Langer et al. [67]	YES: 52% *p* < 0.05	YES: 20% improvement in mobility	Not measured	NA	NCT01993693	Excellent	NA
Taggart et al. [71]	NA	NA	NA	NA	NA	Excellent	YES: 87–95% ease of use, receptive and positive experience
Rigby et al. [55]	NA	NA	NA	YES: Diathermic temperature increase by 3.22–4.45C	NA	Excellent	NA
Best et al. [50]	YES: 3.94 points *p* = 0.002	YES: 2.83 kg improvement in strength *p* = 0.04	Not measured	NA	NCT02466308	Good, n = 3 skin response	YES: 92% use of the device and 72% compliance with study
Langer et al. [51]	YES: 2.5 points *p* < 0.03	Not measured	Not measured	NA	NCT01993693	Excellent	NA
Langer et al. [68]	NA	YES: Power *p* = 0.024, torque *p* = 0.031 and work *p* = 0.031 improvement	NA	YES: Blood lactate reduction *p* = 0.002	NA	Excellent	NA
Langer et al. [69]	NA	NA	NA	YES: Diathermic temperature increase by 12–13C	NA	Excellent	NA
Draper et al. [52]	YES: 1.96 point *p* < 0.001	YES: 505 point function *p* = 0.02, 3.2 N strength *p* = 0.03	Not measured	NA	NCT02083861	Excellent, less than 0.1% reported irritation	YES: 93% study retention
Petterson et al. [64]	YES: 2.61 points *p* < 0.001	Not measured	YES: 2.84 points *p* < 0.01	NA	NCT02135094	Excellent, no reportable	YES: 100% compliance
Draper et al. [70]	YES: 3.33 points *p* < 0.05	YES: 87% improvement	Not measured	NA	NCT04177537	Excellent	YES: 55% able to return to work
Madzia et al. [66]	YES: 2.06–2.96 point *p* < 0.001	YES: 510 point function *p* < 0.001	Not measured	NA	NCT04391842	Excellent	YES: 95% receptive and continuation of treatment
N = 13 Studies, N = 372 subjects	N = 9 Studies significant pain reduction	N = 6 studies significant functional improvement	N = 3 studies significant health improvement	N = 3 studies mechanisms of action measured	N = 7 studies registered	N = 13 studies no adverse events	N = 7 high compliance and treatment receptivity

Numeric Rate of Pain Scale (NRS, 0–10), Visual Analogy Scale (VAS, 0–100 mm), Western Ontario and McMaster Universities Osteoarthritis Index (WOMAC, 0–960), Range of Motion (ROM), Global Rate of Change (GROC)

## Future perspective

**Fig. 6:**
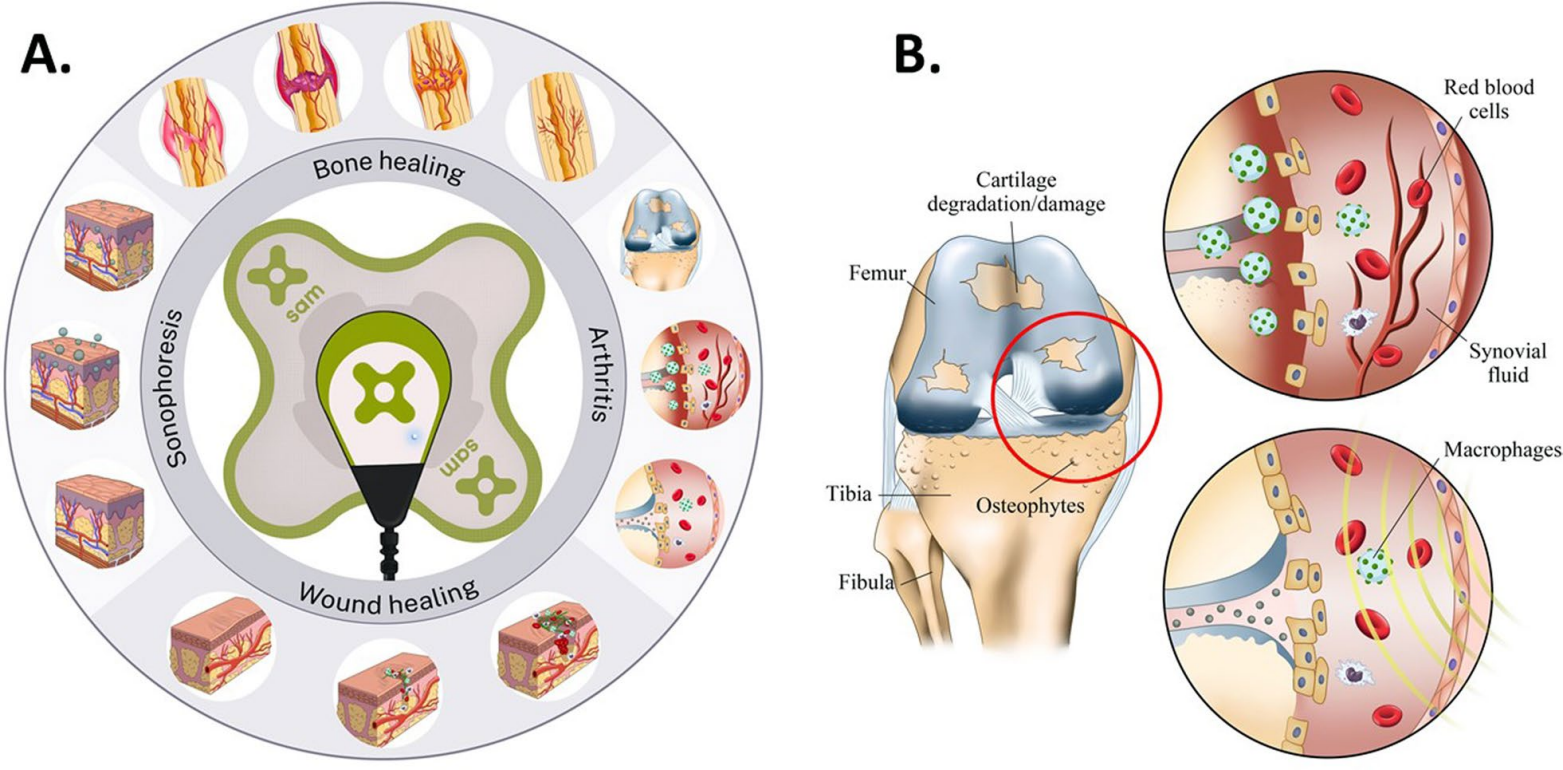
The future application of SAM treatment. **A** Ultrasound thermal and acoustic properties can improve local drug delivery, improve wound and bone healing and reduce arthritis progression. **B** Ultrasound treatment may reduce the progression of osteoarthritis by reducing the rate of osteophyte formation and inflammatory cell activation

SAM has shown excellent results in rehabilitation and pain management, but there are various other potential applications for low-intensity continuous ultrasound (Fig. 6A). This modality has been shown to have chondroprotective effects and slow down the progression of arthritis in clinical studies (Fig. 6B) [79]. More studies are required to understand the underlying mechanism, but it is known that ultrasound inhibits detrimental inflammatory effects on articular cartilage [40, 80, 81]. The FDA has approved low-intensity pulsed ultrasound for non-union fracture healing, and low-intensity continuous ultrasound has a potential to be used in fracture healing as well [82, 83]. The acoustic forces and mechanical stimuli generated by SAM over a longer time course could play a pivotal role in accelerating endochondral ossification, differentiation of chondrocytes based on soft callus into hard classified bone. The acoustic force enhances the differentiation of chondrocytes to bone-forming osteoblast cells and the formation of a calcified collagenous extracellular matrix [84, 85]. Targeted drug delivery remains to be an unmet challenge as well. Ultrasound is used regularly in the clinical setting for topical drug delivery and is considered a viable option [86–88]. The acoustic force and diathermic effects of ultrasound can increase the permeability of skin layers and push through small and large drug molecules. SAM treatment for sonophoresis, specifically for drugs associated with pain reduction, further enhances the pain management of SAM therapy, as shown by Madiza et al. 2020 [66]. Delayed or chronic wound healing due to type I or II diabetes is potentially another area of interest for applying SAM therapy [89–91]. The acoustic force can potentially enhance the blood flow, oxygenation, cellular migration, and formation of new extracellular to close the open wound and expedite the healing process [91].

## Limitations

Although the systematic review focused on Sustained Acoustic Medicine (SAM) for the treatment of musculoskeletal injuries, it is possible that other relevant studies using similar treatment parameters (3 MHz ultrasound at 1.3 W) are available in the scientific literature to further aggregate and synthesize the clinical literature. This limitation was beyond the scope of this research but could be considered in a future analysis paying close attention to time, duration, dose delivered and regularity of ultrasound treatment. The literature search strategy we employed found 13 relevant articles specific to SAM that are more than other past reviews on Sustained Acoustic Medicine ever, it is possible that some relevant studies were missed that were not available in English language or those in the grey literature which are emerging on this new therapeutic treatment [79, 92, 93]. Additionally, several of the outcome variables used in the studies differed in both measure, physical location on the body, condition being treated and control group which limited the scope of meta-analysis. However, we are confident that the most relevant clinical studies on SAM have been identified, and the categorical grouping of the studies supports the inferences drawn.

## Conclusions

This systematic review and meta-analysis reported the current evidence for Sustained Acoustic Medicine on musculoskeletal injuries and chronic pain. SAM, a novel mechanobiological treatment, is clinically effective at reducing pain, improving overall health quality, generating deep therapeutic heat, and increasing mobility leading to a better-quality life and return to daily activities. The prescription home use treatment has excellent safety, usability and satisfaction characteristics for patients, and may be considered a good non-pharmacological and non-invasive treatment option in musculoskeletal injuries.

## Data Availability

All data generated and analyzed during this study are included in this published article.
